# Characterization and Evaluation of Zero-Order Release System Comprising Glycero-(9,10-trioxolane)-trialeate and PLA: Opportunity for Packaging and Biomedicine Applications

**DOI:** 10.3390/polym16243554

**Published:** 2024-12-20

**Authors:** Olga V. Alexeeva, Marina L. Konstantinova, Valentina Siracusa, Vyacheslav V. Podmasterev, Levon Yu. Martirosyan, Olga K. Karyagina, Sergey S. Kozlov, Sergey M. Lomakin, Ilya V. Tretyakov, Tuyara V. Petrova, Alexey L. Iordanskii

**Affiliations:** 1 Emanuel Institute of Biochemical Physics of Russian Academy of Sciences, 119334 Moscow, Russia; kisisinova@yandex.ru (M.L.K.); vpodmasterev@yandex.ru (V.V.P.); levon-agro@mail.ru (L.Y.M.); olgakar07@mail.ru (O.K.K.); sergeykozlov1@gmail.com (S.S.K.); lomakin@sky.chph.ras.ru (S.M.L.); 2 Department of Chemical Science (DSC), University of Catania, Viale A. Doria 6, 95125 Catania, Italy; vsiracus@dmfci.unict.it; 3 N.N. Semenov Federal Research Center for Chemical Physics Russian Academy of Sciences, 119991 Moscow, Russia; tretiakovi.v@yandex.ru (I.V.T.); tuyara.2312@mail.ru (T.V.P.); aljordan08@gmail.com (A.L.I.)

**Keywords:** polylactic acid, films, ozonide, trioleate, drug release

## Abstract

Glycerol-(9,10-trioxolane) trioleate (OTOA) is a promising material that combines good plasticizing properties for PLA with profound antimicrobial activity, which makes it suitable for application in state-of-the-art biomedical and packaging materials with added functionality. On the other hand, application of OTOA in PLA-based antibacterial materials is hindered by a lack of knowledge on kinetics of the OTOA release. In this work, the release of glycero-(9,10-trioxolane) trioleate (OTOA) from PLA films with 50% OTOA content was studied during incubation in normal saline solution, and for the first time, the kinetics of OTOA release from PLA film was evaluated. Morphological, thermal, structural and mechanical properties of the PLA + 50% OTOA films were studied during incubation in normal saline and corresponding OTOA release using differential scanning calorimetry (DSC), X-ray diffraction (XRD), Fourier-transform infrared (FTIR) spectroscopy and mechanical tests. It was confirmed by DSC and XRD that incubation in the saline solution and corresponding OTOA release from PLA film does not lead to significant changes in the structure of the polymer matrix. Thus, the formation of more disturbed α’ crystalline phase of PLA due to partial hydrolysis of amorphous zones and/or most unstable crystallites in the PLA/OTOA semi-crystalline structure was observed. The degree of crystallinity of PLA + OTOA film was also slightly increased at the prolonged stages of OTOA release. PLA + 50% OTOA film retained its strength properties after incubation in normal saline, with a slight increase in the elastic modulus and tensile strength, accompanied by a significant decrease in relative elongation at break. The obtained results showed that PLA + 50% OTOA film could be characterized by sustained OTOA release with the amount of released OTOA exceeding 50% of the initial content in the PLA film. The OTOA release profile was close to zero-order kinetics, which is beneficial in order to provide stable drug release pattern. Developed PLA + 50% OTOA films showed a strong and stable antibacterial effect against *Raoultella terrigena* and *Escherichia coli*, bacterial strains with multidrug resistance behavior. The resulting PLA + OTOA films could be used in a variety of biomedical and packaging applications, including wound dressings and antibacterial food packaging.

## 1. Introduction

The ever-increasing global consumption of plastics leads to an increase in the total volume of packaging materials and industrial waste [1]. Since plastic waste is predominantly non-recyclable and non-biodegradable [2], plastic pollution is a widespread environmental concern due to the severe impact of toxic degradation products on water and soil quality [3]. Therefore, the use of biodegradable plastics based on PLA and other polyesters reflects the ongoing endeavor to solve the global problem of environmental pollution.

During the last decade, various applications of PLA have been proposed, which include a range of biomedical implementations such as tissue engineering and drug delivery, wound dressing and regenerative medicine, as well as diverse usage in agriculture and packaging industry [4,5,6,7,8,9,10]. Previous studies have shown that functionalization with various additives could improve physicochemical properties of PLA and its blends/composites, as well as provide additional functionality for the aforementioned applications [11,12,13,14]. Thus, the incorporation of antimicrobial agents (e.g., OTOA, cinnamaldehyde, etc.) into the PLA matrix can impart antibacterial properties to the modified material, preventing the growth of microorganisms inside the package [15,16]. This leads to the concept of antimicrobial active packaging, which is considered to be promising in inhibiting the growth of the microorganisms in foods while maintaining their quality and safety [8,9,10,17,18]. Simultaneously, the use of biodegradable PLA-based systems with antimicrobial additives could provide enhancement, through plasticizing, of mechanical and diffusional characteristics of packaging materials [14,19,20]. Another important possible application of functionalized PLA-based materials is high-tech wound dressings, which are gradually replacing gauze dressings in medicine [21,22,23]. The introduction of an antibacterial agent into wound-dressing materials gives additional functionality and meets modern requirements for the prevention of secondary infections [24,25].

Currently, ongoing efforts are being made for the development of novel PLA-based materials with added antibacterial functionality, which could assure reliable protection against microbial impact on food products and medical devices [26,27,28]. In this connection, the request immediately arises about the impactful release of active antibacterial agents incorporated in polymer matrices into aqueous media. The latter is one of the most essential properties of antibacterial polymer-based materials, since it is necessary to ensure desirable release kinetics (e.g., avoid large burst release) and provide long-term bioavailability of the active agent [29,30]. The release kinetics of an active agent from a biomaterial matrix is affected by a combination of several complex factors, including matrix properties, active agent loading and active agent–matrix interactions [31,32]. In water-containing media, the sustained release of antibacterial agents could be accompanied by macromolecular hydrolysis [33], as well as by dissolution and erosion of the polymer matrix [34,35,36]. Previously, the phenomena related to drug release from biodegradable polyesters, such as polyhydroxybutyrate (PHB) and PLA, formulated as films and ultrathin electrospun fibers, were extensively studied and modeled [37,38]. A detailed study of changes in physicochemical properties of PLA-based materials with added antibacterial functionality during the release of the active agent, together with release kinetics, could establish the specific factors which influence the relations between the matrix properties and antibacterial agent release process.

According to our previous works, glycero-(9,10-trioxolane)-trioleate (ozonide of oleic acid triglyceride, OTOA), the product of ozonation of natural vegetable oils, could be used as a plasticizer and functional additive for various PLA materials (films, fiber mats) [6,11]. OTOA is non-toxic, biocompatible and possesses good antimicrobial activity. It has been shown recently that the introduction of OTOA into the polylactic acid/polycaprolactone (PLA/PCL) films leads to significant enhancement in their physicochemical properties and provides additional antimicrobial functionality for various applications [13]. At the same time, effective application of OTOA-loaded PLA materials is hindered by a lack of data on the OTOA release from PLA films.

In this work, for the first time, the kinetics of OTOA release from the PLA film with 50% OTOA content into the aqueous media was studied. The evolution of physicochemical, morphological and mechanical properties of OTOA-loaded PLA films during the OTOA release process were studied by DSC, XRD, FTIR spectroscopy and mechanical tests. In addition, the antibacterial activity of OTOA-loaded PLA materials on the *E. coli* and *R. terrigena* (*Klebsiella terrigena*) bacteria was assessed. The findings from the present study could help to establish the relationship between the physicochemical properties of PLA matrix and the OTOA release process and provide a rational design of OTOA-loaded PLA materials with the sustained release of the antibacterial agent for biomedical and packaging applications.

## 2. Materials and Methods

### 2.1. Materials

NatureWorks Ingeo 3801X Injection Grade PLA (SONGHAN Plastics Technology Co., Ltd., Shanghai, China) with the average molecular weight of 1.9 × 10^5^ g/mol was used to obtain PLA and PLA+ 50% OTOA films. The polydispersity index (PDI) for PLA used in this study was about 1.8, which was determined as described previously [39]. Glycero-(9,10-trioxolane)-trioleate (OTOA) was obtained from Medozon (Moscow, Russia), with a previously described chemical structure [11]. To prepare PLA solutions, dry purified chloroform (≥99.5%, Sigma-Aldrich Inc., St. Louis, MO, USA) was used. All reagents were used as received.

### 2.2. Preparation of Films

PLA film materials were prepared by solvent evaporation from chloroform solutions as described previously [11]. For the reference film, PLA (1 g) was dissolved in 50 mL of chloroform. For the PLA + 50% OTOA film, equal amounts of OTOA and PLA were taken (total amount of solutes was 1 g) and further dissolved in 50 mL of chloroform. The resulting solutions were continuously stirred for 12 h. The obtained solutions were poured onto glass plates and dried at room temperature (T = 22 °C) to constant weight. The resulting thicknesses of the films used in the experiments were in the range of 120–140 µm.

### 2.3. OTOA Release Studies

OTOA release from PLA films was studied by incubating the film samples in the normal saline solution (0.9% aqueous sodium chloride (NaCl) solution). All OTOA release experiments were performed using the total immersion method with a known amount of film sample immersed in a sealed vial containing 30 mL saline and incubated in a shaker at a constant incubation temperature of 37 °C.

At certain time points, 2 mL of release medium was removed from the vial, and the absorption spectra were recorded in the range of 200–300 nm using a Shimadzu UV-2600i UV-Vis spectrophotometer (Shimadzu Europa GmbH, Duisburg, Germany). After measurements, the release medium was returned to the vial to maintain the identical release conditions. A linear calibration curve was constructed based on OTOA saline solutions with concentrations from 0 to 3.3 × 10^−3^ mol/L. Specific absorbance at 266 nm related to OTOA for each time point was correlated with OTOA concentration using the obtained calibration curve, and the extinction coefficient of 85 L/(mol∙cm) was estimated. Data on cumulative OTOA release were obtained as the average for three PLA + OTOA film samples and were normalized by the actual initial amount of OTOA in the films. The data on cumulative OTOA release was further used for the mathematical modeling of the OTOA release kinetics. Reference PLA film samples were incubated in a similar manner to determine the background from the PLA films without OTOA and obtain reference PLA films after incubation for various physicochemical studies. In addition, water sorption for PLA and PLA + OTOA films was studied as described previously [13].

#### Analysis of Drug Release Data Using Mathematical Models

The application of drug release data to mathematical models is usually made using various mathematical equations that define the drug dissolution profile depending on the properties of the release system, its geometry, drug–matrix interaction, etc. Once a suitable function has been determined, the obtained drug release profile can be correlated with a particular drug release kinetic model. Regularly, mathematical modeling allows the experts to determine the mechanism of drug release, which can change from anomalous mass transport being due to polymer segmental relaxation to proper diffusion. In addition, mathematical modeling allows the measurement of some important physico-chemical parameters, such as the drug diffusion coefficient in the polymer matrix.

To assess possible mechanisms of OTOA release from the PLA matrix, the experimental data were fitted to phenomenologically relevant models. Two utmost mechanisms of drug release from therapeutic systems are outlined in the literature. One of them is considered exceptionally as diffusive, i.e., the drug output from the polymer vehicle is carried out through segmental mobility and/or specific free volume of the polymer system. In this case, a drug release profile is determined by two equations. For the initial period of drug release, that is at the condition of *C_t_/C_∞_* ≤ 0.5 and at a constant diffusion coefficient *D*, it is correct to present [40,41]

*C_t_/C_∞_* = (8*/*π^1/2^)·[*D*·*t*·*L*^−2^]^1/2^, (1)

where *C_t_* and *C_∞_* are released concentration at moment *t* and *t* → ∞, *L* is the thickness of the slab containing the drug. At initial uniform distribution of the drug in the slab and for the final stage of its delivery (*C_t_/C_∞_* ≥ 0.5), the kinetic profiles are described by the semilogarithmic expression [40]:

log[1 − (*C_t_/C_∞_*)] = log(8/π^1/2^) − π^2^·*D*·*t*/*L*^2^, (2)

where all symbols are the same as in Equation (1).

The alternative description of drug release is provided by the more general Korsmeyer–Peppas equation [42]:

*C_t_/C_∞_* = *k·t^n^*, (3)

where *C_t_/C_∞_* is the fraction of drug released at time *t*, *k* is the release rate constant, and *n* is the release exponent which is indicative of the transport mechanism. In the case of thin films with negligible edge effects, Fickian diffusion is characterized by *n* = l/2 [41]. Drug release determined by the polymer relaxation only without the impact of diffusion, which is more typical for rigid polymers below glass transition temperature such as PVA, PMMA and PLA, as well as the glassy-state gels, provides zero-order release kinetics with the exponent *n* = 1 [41,43]. The experimental data were fitted using the models described above.

### 2.4. Morphology and Opacity of PLA Films

The surface morphology of PLA and PLA + OTOA films before and after incubation was studied using an OLIMPUS CX21 optical microscope (Olympus Corp., Tokyo, Japan) and then processed using MICAM 3.02 software.

The opacity of PLA films was probed using a Shimadzu UV-3600 spectrophotometer (Shimadzu Europa GmbH, Duisburg, Germany) and was calculated with the following equation:

Opacity (mm^−1^) = *A*_600_/*X*, (4)

where *A*_600_ is the absorbance of the film at 600 nm and *X* is the thickness of film sample (mm) [44]. The thickness of the PLA films was measured using the digital micrometer and presented as the average value ± SD for at least 10 measurements at different sites on the film.

### 2.5. FTIR Spectroscopy Measurements

FTIR spectra of PLA films before and after incubation were obtained as described previously [6,13]. Briefly, a Bruker Tensor 27 IR Fourier spectrometer (Bruker Corporation, Billerica, MA, USA) was used, equipped with a PIKE MIRacle ATR accessory with a Teflon cell and germanium crystal (PIKE Technologies, Madison, WI, USA), which allows the measurements of solid samples. PLA film samples were tightly pressed to the surface of the Ge crystal in order to ensure good optical contact. FTIR spectra were recorded in the 4000–400 cm^−1^ range with 4 cm^−1^ resolution using an average of 16 consecutive scans.

### 2.6. X-Ray Diffraction Analysis

PLA and PLA + OTOA films before and after incubation were studied by XRD using a DRON-3M X-ray diffractometer (Burevestnik, St. Petersburg, Russia) in the 2*θ* range of 10–40° as described previously [6]. Relative crystallinity of the films was estimated as

*χ* = *I_C_*/(*I_C_* + *I_A_*) (5)

where *I_A_* and *I_C_* are the integral intensities corresponding to the respective amorphous and crystalline phases [45]. The relative error for *χ* (XRD) determination does not exceed 5%.

### 2.7. Differential Scanning Calorimetry 

Thermal properties of the PLA and PLA + OTOA films before and after incubation were studied using a Netzsch DSC 204 F1 Phoenix differential scanning calorimeter (Netzsch, Selb, Germany) in inert Ar atmosphere. PLA film samples with the weight of ~5 mg were placed in aluminum pans and heated from 20 °C to 200 °C at 10 °C/min rate. Indium, tin and lead were used to calibrate the instrument. Due to the high exothermic effect associated with the decomposition of OTOA and subsequent volatilization of its decomposition products, only one heating stage was performed in DSC experiments in order to characterize the thermal properties of originally obtained PLA films without “erasing their thermal memory”.

The degree of crystallinity (*χ*) for the studied films was calculated according to the following equation, assuming no cold crystallization took place during heating:

(6) $$\chi =\frac{\Delta H_{m}}{\Delta H_{m}^{100}\times \left(1-\beta \right)}\times 100\%$$

where Δ*H_m_* is the experimental melting enthalpy; Δ*H_m_*^100^—theoretical melting enthalpy of the 100–crystalline PLA (93.6 J/g); *β*—mass fraction of OTOA additive in the film. Deconvolution of complex DSC peaks obtained for the PLA + OTOA films was performed using NETZSCH Peak Separation 2006.01 program employing the Fraser–Suzuki algorithm for asymmetric DSC curves, as was described previously [6,13].

### 2.8. Mechanical Properties of PLA Films

Mechanical tests of PLA films before and after incubation were performed using the Zwick Z010 testing machine (ZwickRoell GmbH & Co., Ulm, Germany) at room temperature. The layout of the samples used in the mechanical tests was given previously [6].

Loading diagrams (load F vs. deformation ԑ) were obtained at a loading speed of 1 mm/min. Mechanical parameters of the films (elastic modulus E, tensile strength σ and relative elongation at break) were determined from the loading diagrams. Five samples of each PLA film type (reference PLA and PLA + OTOA films) were tested before and after exposure to saline and OTOA release. Results are presented as mean ± standard deviation at a significance level of *p* < 0.05.

### 2.9. Measurements of Antibacterial Activity

The antibacterial activity of PLA and PLA + OTOA film samples against two bacterial strains with possible multidrug resistance was measured by the Murray paper disk method [46,47]. *Raoultella terrigena* (Klebsiella terrigena) and *Escherichia coli* strains from the Korean Cell Line Bank were used in the experiments. Then, 100 μL of the culture medium of each strain was evenly spread on TSA (tryptic soy agar). After adding bacteria to the TSA medium, the culture was enriched for 18–24 h at 35–37 °C.

The inoculum was prepared from an 18–20 h agar culture in meat peptone broth, bringing the turbidity to 0.5 McFarland standard. The resulting broth culture was diluted 10 times with a sterile isotonic NaCl solution, which corresponded to a final concentration of around 1 × 10^7^ CFU/mL. Inoculum was applied to Petri dishes with a dense nutrient medium using sterile cotton swabs. Thereafter, disks (6.0 ± 0.1 mm in diameter) were cut from PLA and PLA + OTOA films and applied to the seeded surface using sterile tweezers. Afterwards, the dishes were incubated for about 20 h at 37 °C. At the end of incubation, the retention of the visible growth zone was calculated based on complete inhibition of visible growth [48,49]. The experiment was repeated in triplicate.

## 3. Results and Discussion

### 3.1. OTOA Release from PLA Films

**Figure 1:**
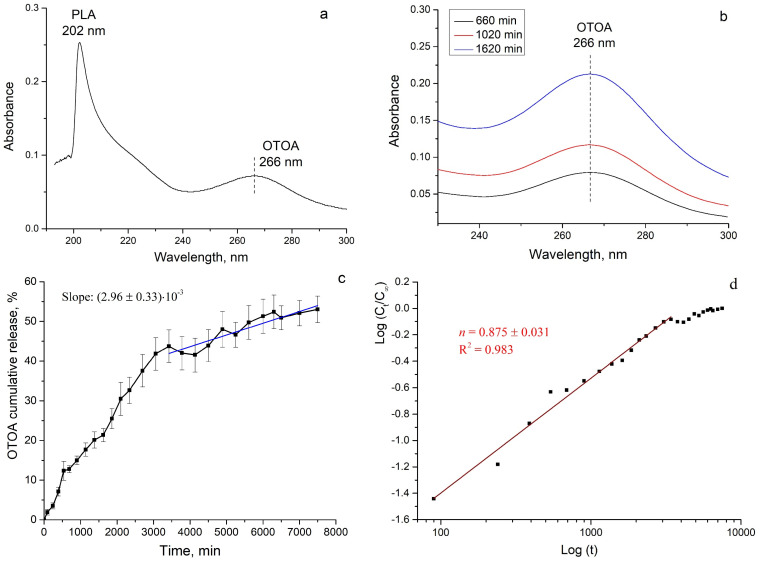
(**a**) UV-Vis spectrum of the release medium obtained during PLA + 50% OTOA film incubation; (**b**) absorption peak at 266 nm related to OTOA; (**c**) cumulative OTOA release kinetics from the PLA + 50% OTOA film; (**d**) approximation of the cumulative OTOA release kinetics using Equation (7).

The measurement of OTOA release from PLA films was carried out by UV spectroscopy. The UV-Vis spectrum of the release medium obtained during incubation of PLA + 50% OTOA film is shown in Figure 1a. The absorption band at about 202 nm is related to PLA, whereas the absorption peak at 266 nm could be related to OTOA. The intensity of the latter band is continuously increased in the course of OTOA release from the PLA film (Figure 1b). Such a correlation allows the authors to obtain a calibration curve for the evaluation of the OTOA release kinetics from the PLA film.

The corresponding curve of cumulative OTOA release from PLA + 50% OTOA films into aqueous NaCl solution is presented in Figure 1c. To examine the release kinetics, the logarithmic form of Equation (3) was applied to the obtained results and the value of the release exponent *n* was obtained by linear fit:

log_10_ (*C_t_/C_∞_*) = log_10_ *k* + *n*∙log_10_
*t* (7)

It should be noted that the above mathematical model is only valid for the first 60% of the cumulative release [41]. As can be seen from Figure 1d, the kinetic profile of OTOA release has two distinct stages. Thus, the initial OTOA release period up to 3000 min exhibited an *n* value of 0.875 ± 0.031. The second stage above 3000 min shows an extremely low rate of OTOA release.

Drug-loaded PLA-based materials can show different release mechanisms, including the dissolution of drug molecules, permeation of fluid into polymer material followed by the diffusion of the molecules and polymer degradation/erosion. OTOA release during the first stage, characterized by a release exponent value of 0.875, could be attributed to the case of anomalous transport, in which drug delivery is due to both diffusion and relaxation of the polymer matrix. As the *n* value is close to 1, it could be deduced that the OTOA release process is substantially controlled by swelling and relaxation of the PLA matrix (case-II transport).

The low rate of OTOA release at a later stage (longer than 3000 min) appears to be associated with the slow degradation of the PLA polymer. As PLA is slowly swellable, OTOA dispersed in the PLA matrix would be released mostly when PLA degrades. The possible mechanisms are the dissolution of OTOA molecules on the PLA surface, when pores and voids are created in the PLA matrix, and diffusion of OTOA molecules near the film surface. The decrease in the OTOA release rate at the second stage demonstrates limited PLA degradation within the studied period. Therefore, the second stage of OTOA release could be approximated by the zero-order kinetics with the zero-order rate constant of (2.96 ± 0.33)∙10^−3^ h^−1^.

The reasons for the zero-order release mechanism have been discussed in many previous publications. For biodegradable polymers, hydrolysis and enzymatic degradation occurring through a surface-erodible moving front show zero-order kinetics [50]. The nature of the drug, the hydrophobicity degree of its molecular structure, opens the possibility to vary the character of drug release. For example, hydrophobic formulation of bupivacaine was released in accordance with zero-order mechanisms, at least in the initial stage of delivery, while its hydrophilic formulation as bupivacaine hydrochloride showed first-order drug release [51,52]. In addition, it is well known that for the glassy-state polymers, the zero-order release of the low molecular compounds has been explained by the interplay between diffusional and relaxation processes leading to the so-called Case II mechanism [53]. For instance, many samples of PLA prepared in the forms of particles demonstrate the same zero-order kinetics for injectable microparticles [54]. Here, it is worth noting that, in 1989, the FDA recognized PLA and PLGA as “a long-acting drug delivery depot” [51,52]. The release profile could also be modified by coating, as was shown in [55]. Zero-order release has been shown for metoprolol and propranolol from the pellets comprising microcrystalline cellulose and coated by polyvinyl acetate layers. In the absence of coating, the same pellets do not provide the characteristics of the zero-order drug delivery systems.

Zero-order drug release kinetics eliminates the flaws related to the non-linearity of delivery supported by the diffusion mechanism via the square-root equation (Equation (1)) and the logarithmic equation (Equation (2)) and provides the drug release with a constant rate. A stable release pattern is efficiently designed to save expensive pharmaceutics, to keep the drug content within the therapeutic window, to decrease dosing frequency and to enhance the patient’s adherence to the pharmaceutic treatment [52].

### 3.2. Morphology of PLA Films

**Figure 2:**
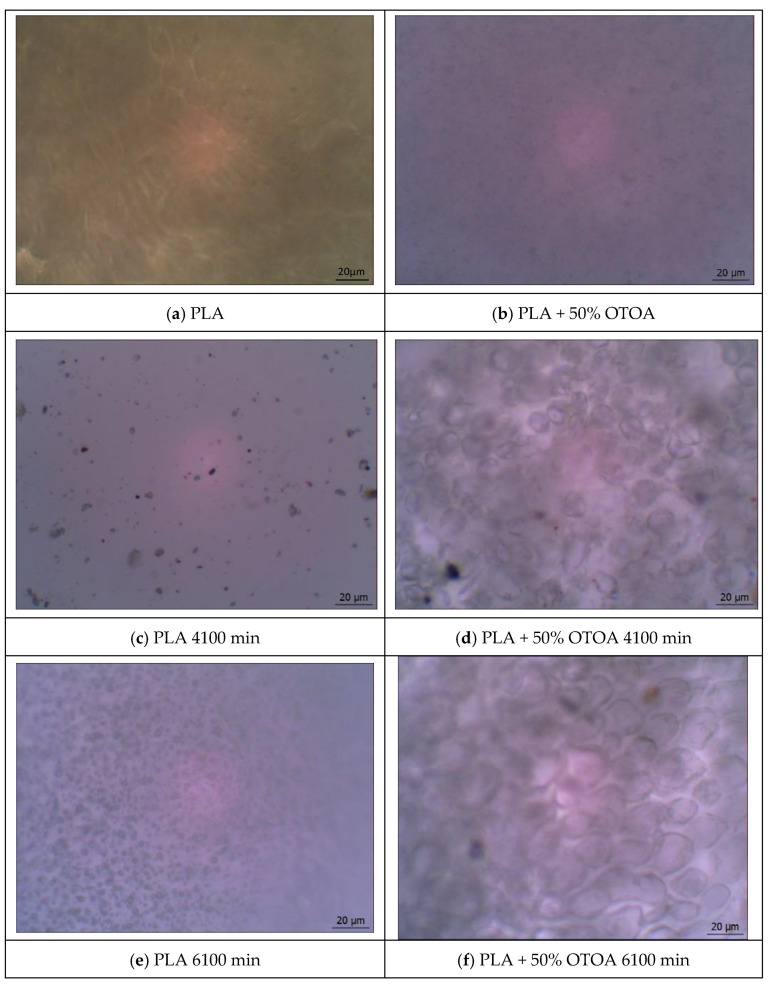
Optical microphotographs of the pristine PLA (**a**) and PLA + 50% OTOA (**b**) films; optical microphotographs of PLA (**c**,**e**) and PLA + 50% OTOA (**d**,**f**) films after different exposure times in saline solution.

Recently, the authors have shown that the introduction of a modifier agent like OTOA significantly changes the appearance and morphology of PLA films, as well as their bulk structure and surface properties [6,11]. Figure 2 shows the optical images of the pristine PLA and PLA + 50% OTOA films, as well as their photographs after soaking in saline solution at different exposure times, namely, at 4100 and 6100 min. According to the data provided above (see Section 3.1), these exposure times correspond to the beginning and the end of the second stage of OTOA release. It is well established that PLA, as a crystallizable polymer, has spherulitic morphology due to isothermal crystallization in the polymer solution. During crystallization in the presence of OTOA, the PLA molecules are oriented relative to a common center, but they are significantly moved apart by OTOA as a plasticizer. For steric reasons, OTOA is predominantly located in the inter-crystalline space in the volume of the PLA film [6].

Taking into account the relatively short times of incubation in NaCl solution (6100 min), large hydrolytic changes in the studied films were not expected [56,57]. As can be seen from Figure 1, minor hydrolytic changes were observed for the reference PLA film, which could be characterized by a smooth surface without visual defects. In contrast, optical microscopy clearly shows the morphological changes for the PLA + 50% OTOA film depending on the exposure time in physiological saline. These changes become more pronounced in the course of the OTOA release (Figure 1). It could be proposed that the release of OTOA occurs from the inter-spherulitic space and does not affect the intra-spherulitic interactions of OTOA and PLA, leading to a clear contrast between intra- and inter-crystalline areas of PLA + 50% OTOA film.

Transparency is an important physical property of polymer films, which is always directly related to their composition, crystallinity and the chemical processes occurring inside the material. Visually, both PLA and PLA + 50% OTOA films became cloudy and non-transparent after incubation in the NaCl solution, as compared to the pristine films. The opacity of the films was measured spectrophotometrically at a wavelength of 600 nm and calculated using Equation (4) (Table 1).

As could be seen, an increase in opacity in the course of incubation was observed for both film types. On the other hand, pristine PLA + 50% OTOA film showed increased opacity due to the presence of the OTOA itself, which could penetrate both into the inter- and intra-spherulitic space of the polymer material. After soaking the PLA + 50% OTOA film in saline, an even greater increase in opacity was observed, which indicates that slow hydrolytic processes in the film take place as OTOA is released from the PLA matrix.

### 3.3. FTIR Spectroscopy

**Figure 3:**
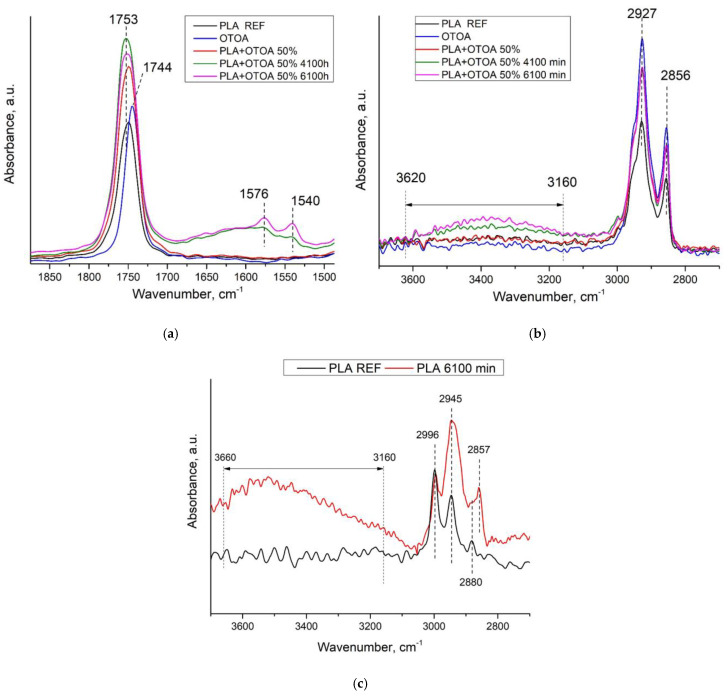
FTIR spectra of pure OTOA, pristine reference PLA and PLA + 50% OTOA films, PLA + 50% OTOA films after different exposure times in saline solution: (**a**) close-up view of FTIR spectra at 1500–1850 cm^−1^ interval; (**b**) FTIR spectra at 2800–3600 cm^−1^ interval; (**c**) FTIR spectra of reference PLA film before and after 6100 min incubation in NaCl solution.

We considered the features of the chemical interaction of PLA and OTOA in a previous study, in which the FTIR spectra of PLA films with different OTOA contents were obtained [6]. Here, in the spectra of the pristine PLA film, characteristic bands are observed at 1455 cm^−1^ and 1753 cm^−1^ (Figure 3a), resulting from the bending vibrations of -CH_3_ and stretching vibrations of the C=O group, respectively, as well as absorption bands at 2944 cm^−1^ and 2995 cm^−1^ (Figure 3b), which are assigned to the asymmetric stretching vibrations of the C-H group [58,59]. Table 2 shows the characteristic bands observed in the FTIR spectra for the studied reference PLA and PLA + 50% OTOA films and their assignment.

The process of incubation of reference PLA films in normal saline solution was accompanied by a slow hydrolytic process, as indicated by the FTIR spectra (Figure 3c). An increase in the intensity of the 2945 cm^−1^ band assigned to C-H stretching of the -CH_3_ group was observed, which indicates the formation of additional -CH_3_ groups during incubation in saline solution. In addition, a band at 2857 cm^−1^ appeared in the spectrum, possibly as a consequence of the formation of -CH_2_-Cl groups instead of -CH_3_ groups, since the exposure was carried out in an aqueous NaCl solution. The appearance of a characteristic broad band related to -OH groups was also observed in the 3160–3620 cm^−1^ range.

**Figure 4:**
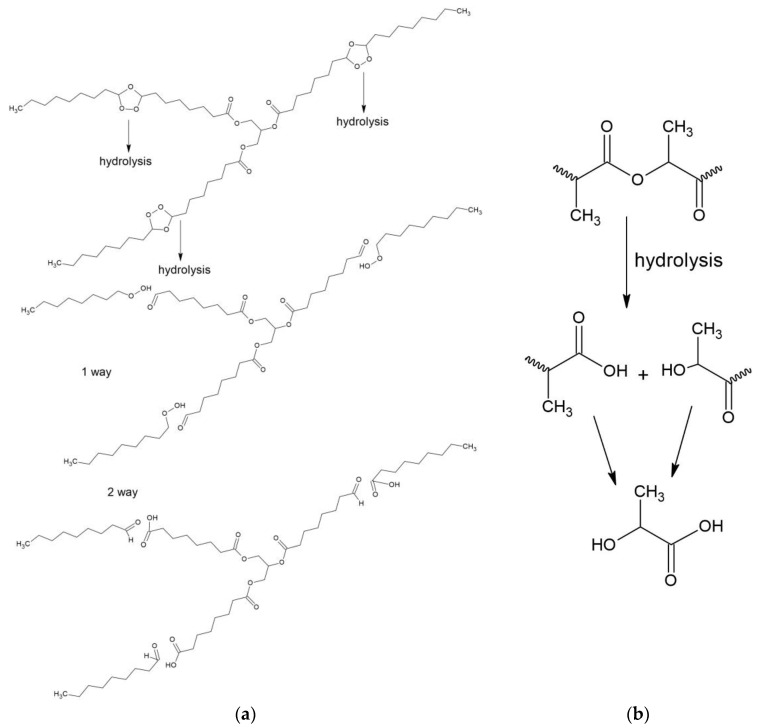
Hydrolysis scheme of OTOA (**a**) and PLA (**b**).

In the spectra of PLA + 50% OTOA films, two additional bands appeared at 2927 cm^−1^ and 2856 cm^−1^, which could be attributed to symmetric and asymmetric stretching vibrations of -CH_2_ groups [60,61]. As PLA does not contain -CH_2_ groups in its chemical structure, the presence of these bands in the FTIR spectra of PLA + 50% OTOA films is evidence of the encapsulation of OTOA in the PLA film volume, since -CH_2_ groups are abundant in OTOA only. The obtained FTIR spectra could help to establish whether the OTOA release is predominantly accompanied by PLA hydrolysis or OTOA degradation in the framework of the schemes given in Figure 4.

As can be seen from the spectra presented in Figure 3a,b, the process of OTOA release from the PLA matrix is also accompanied by partial destruction of the ozonide structure of OTOA with the formation of acidic -COO groups, which is indicated by the appearance of additional bands at 1540 and 1576 cm^−1^, and an increase in the intensity of characteristic bands at 3160–3620 cm^−1^ related to -OH groups [62]. The increase in the intensity of these bands correlates with an increase in incubation time. One can also observe an increase in the intensity of the 1750 cm^−1^ band in the course of incubation, which could be assigned to C=O stretching vibrations. Based on the analysis of FTIR spectra, it could be assumed that both the PLA matrix and OTOA molecules undergo slow hydrolysis while soaking in the saline solution; however, these processes are apparent only at the prolonged stage of incubation and do not affect the general kinetics of OTOA release.

### 3.4. XRD

**Figure 5:**
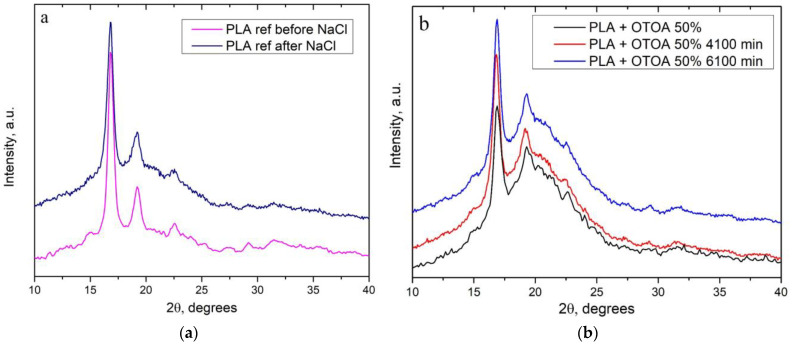
XRD patterns of (**a**) reference PLA films before and after incubation in NaCl solution, and (**b**) PLA + 50% OTOA films before and after incubation in NaCl solution for 4100 min and 6100 min.

Incubation in the NaCl solution and/or OTOA release from the film under the influence of diffusion–hydrolytic processes could affect the structural characteristics of PLA films [63]. Therefore, crystalline characteristics of reference PLA and PLA + 50% OTOA films before and after incubation in NaCl solution were examined by XRD. The corresponding XRD diffractograms are shown in Figure 5.

Pristine PLA film (prior to NaCl incubation) showed characteristic diffraction peaks at 16.8°, 19.2° and 22.6° 2θ, confirming the presence of PLA crystalline structures in the film (Figure 5a). Intense peaks at 16.8° and 19.2° could be related to the diffractions of (200)/(110) and (203) planes, while smaller peaks at 22.6° and 24.1° correspond to (210) and (213) diffractions, respectively [64]. According to previous reports, the presence of both (210) and (213) diffractions in the XRD pattern points to the existence of the PLA α phase in the PLA film [65]. It could be concluded that for the pristine PLA film, a combination of the α phase and α’ phase was observed. The degree of crystallinity for the reference PLA film prior to NaCl incubation was estimated as 35.5% (Table 3), which is consistent with our previous findings [6]. As can be seen from Figure 5a, incubation in the NaCl solution led to minor changes in the structural properties of the reference PLA film, which showed the same characteristic diffraction peaks at 16.8°, 19.2° and 22.6° 2θ, as were observed for the pristine PLA film prior to incubation. The absence of the (213) reflection at 24.1° for the NaCl-incubated PLA film is evidence of the transformation of the PLA α crystals to the α’ form, which could be accounted for by the effect of the hydrolytic processes. In addition, incubation in the NaCl solution led to an increase in the contribution of the amorphous phase (amorphous halo), corresponding to the amorphous regions of the semi-crystalline PLA structure. The latter was confirmed by the decrease in the degree of crystallinity for the reference PLA film to 31.3% after incubation (Table 3).

All studied PLA + 50% OTOA samples showed intense diffraction peaks at 16.2° and 19.2° 2θ, together with a weak peak at 22.6° 2θ (Figure 5b). A small peak corresponding to the (210) diffraction and the absence of the (213) reflection suggest that the α’ phase was the primary crystalline phase for all studied PLA + 50% OTOA films. The degree of crystallinity for the pristine PLA + 50% OTOA film (prior to NaCl incubation) was 24.3% (Table 3), which is similar to the values observed previously [6,13]. As compared to the reference PLA films, a significant increase in the contribution of the amorphous phase was observed for all studied PLA + 50% OTOA films, which could be accounted for by the formation of the OTOA phase and/or inclusion of OTOA in the amorphous regions of the semi-crystalline PLA structure [6]. Additionally, the intensity of the diffraction peak at 22.6° 2θ was decreased for PLA + 50% OTOA films after incubation in NaCl solution and OTOA release. This could be attributed to the formation of more disturbed α’ phase as a result of incubation.

On the other hand, the intensity of the (200)/(110) reflection at 16.8° was significantly increased for both NaCl-incubated PLA + OTOA films (Figure 5b), as compared to the pristine PLA + 50% OTOA film prior to incubation. The latter was accompanied by an increase in the degree of crystallinity for the NaCl-incubated PLA + OTOA films, as shown in Table 3. It could be deduced that the hydrolysis of amorphous zones in the PLA semi-crystalline structure and OTOA release take place simultaneously in PLA + OTOA films during incubation. Both processes increase the crystalline-to-amorphous regions ratio, leading to the increased degree of crystallinity [66]. Moreover, hydrolyzed amorphous PLA could form additional crystalline structures due to the enhanced mobility of polymer chains, as was manifested by the increase in the intensity of the (200)/(110) diffraction for saline-incubated PLA + OTOA films [67].

### 3.5. DSC

**Figure 6:**
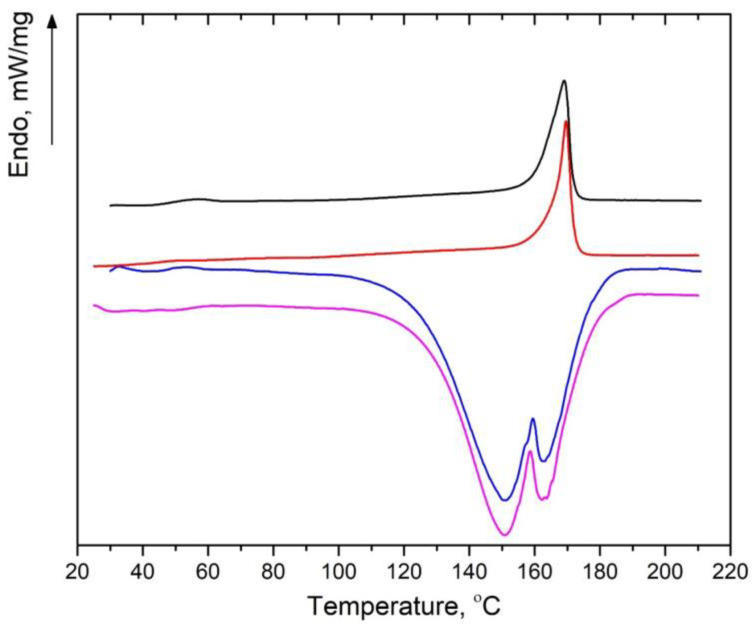
DSC thermograms of the reference PLA films prior to (black) and after incubation in NaCl solution (red), pristine PLA + 50% OTOA film (blue) and PLA + 50% OTOA film after incubation for 6100 min (magenta).

Figure 6 shows the DSC curves for the reference PLA films prior to and after incubation in NaCl solution for 6100 min, as well as for pristine PLA + 50% OTOA film and PLA + 50% OTOA film after incubation for 6100 min. DSC thermograms for the reference PLA films show a single endothermic peak, which could be attributed to PLA melting [64,67,68]. No traces of the cold crystallization were observed for the studied reference films. As can be seen from Table 4, the T_m_ value for the reference PLA film was somewhat increased after incubation. Additionally, the PLA melting peak became significantly narrower after incubation. Both findings indicate that reference PLA films after incubation possess more stable crystalline structures as compared to the pristine one. The values of the melting enthalpy (Δ*H_m_*), as well as the degree of crystallinity (*χ*) of the reference PLA films, calculated according to Equation (6), are shown in Table 4. As seen, incubation in NaCl solution leads to a minor decrease in the melting enthalpy and, consequently, in the degree of crystallinity of the pristine PLA film, which shows good correlation with the XRD data. The obtained results evidence that incubation leads to partial hydrolysis of the amorphous zones and/or most unstable crystallites in the reference PLA film, which leads to the corresponding decrease in the degree of crystallinity.

DSC curves for the PLA + 50% OTOA films show superposition of exo- and endothermic peaks in the temperature range of 120–180 °C. The endothermic peak corresponds to PLA melting, whereas the exothermic one is due to a complex irreversible process of OTOA thermal destruction, which involves breaking the C-O-O-C bonds and the formation of C-OH groups [6].

Deconvolution of the overlapping exo- and endothermic calorimetric peaks made it possible to estimate the melting enthalpy of PLA and determine the degree of crystallinity for the studied PLA + OTOA films (Table 4).

As could be seen, the melting enthalpy and the degree of crystallinity were significantly increased for the PLA + 50% OTOA film as a result of incubation, whereas the melting temperature was decreased. The results obtained by DSC show good correlation with the XRD data and could be accounted for by the two simultaneous processes taking place in the PLA + 50% OTOA films during incubation in the NaCl solution, i.e., OTOA release and the hydrolysis of amorphous zones and/or most unstable crystallites in the PLA/OTOA semi-crystalline structure. As a result, a more disturbed crystalline phase forms as a result of incubation, leading to a decrease in the melting temperature of PLA. On the other hand, the partial hydrolysis of PLA amorphous zones could lead to enhanced mobility of hydrolyzed PLA chains, which could reorganize and form additional crystalline structures. This was manifested by an increase in the degree of crystallinity obtained by DSC and an increase in the intensity of the (200)/(110) reflection as shown by XRD.

### 3.6. Mechanical Properties of the Films

**Figure 7:**
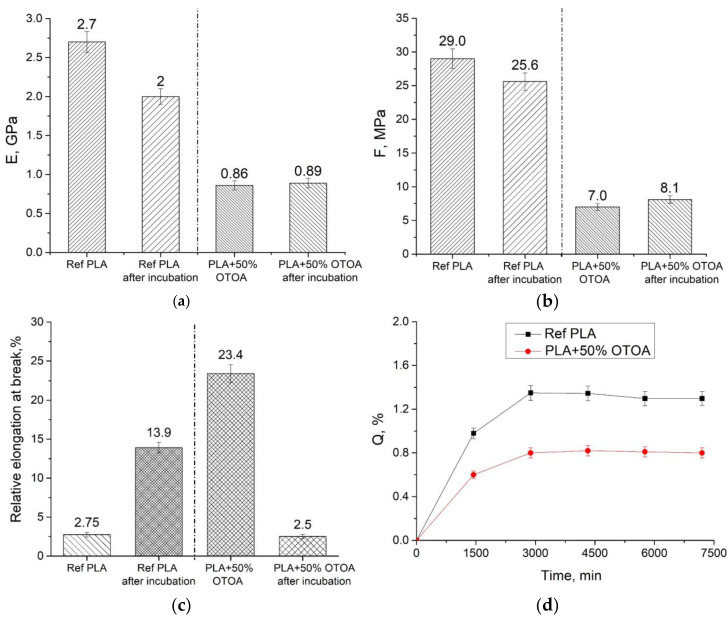
Elastic modulus (**a**), tensile strength (**b**) and relative elongation at break (**c**) for pristine PLA and PLA + 50% OTOA films and respective materials after incubation in normal saline for 6100 min. Sorption capacity (Q) of PLA and PLA + 50% OTOA films during incubation in water (**d**).

The cumulative release of more than 50% OTOA from the PLA + OTOA film during incubation in the saline solution could certainly affect the mechanical properties of the material. The elastic modulus (E), tensile strength (σ) and elongation at break were evaluated for PLA and PLA + 50% OTOA films prior to and after incubation in NaCl solution for 6100 min (Figure 7).

As can be seen from Figure 7, the reference PLA sample after exposure in normal saline solution for 6100 min showed decreased tensile strength and elastic modulus and increased elongation at break. Both trends are apparently due to swelling and increased mobility of polymer chains in the PLA matrix as a result of partial PLA hydrolysis. Figure 7d shows the water sorption of PLA films during incubation in distilled water. As expected, the reference PLA film showed limited swelling during incubation in water, while the PLA + OTOA film showed an even lower degree of swelling due to the hydrophobic nature of OTOA. Therefore, we assume that the swelling of PLA films does not significantly influence the mechanical properties of PLA films. In addition, the diffusion of OTOA through the polymer matrix due to the swelling of PLA + OTOA film would be limited. According to the results of our previous studies, high OTOA content of more than 50% in PLA films leads to a decrease in the strength characteristics, mostly due to a decrease in the degree of crystallinity and an increase in the content of the plasticizing agent (OTOA) in the PLA matrix [6,69,70]. In the present case, incubation in the saline solution and OTOA release from the PLA matrix leads to a slight increase in the elastic modulus and tensile strength by 3.5% and 8%, respectively (Figure 7). Relative elongation at break for the PLA + 50% OTOA film was drastically decreased after incubation and was close to the corresponding value for the untreated reference PLA sample (2.5%). The observed results could be accounted for by the loss of OTOA, which acts as the plasticizing agent, from the PLA matrix, due to OTOA release into the aqueous medium and partial OTOA hydrolysis.

### 3.7. Antibacterial Activity of the PLA + 50% OTOA Films

The antimicrobial activity of OTOA was measured using the paper disk method for *Raoultella terrigena* (*Klebsiella terrigena*) and *Escherichia coli* bacterial strains. The choice of bacteria was stipulated by their prevalence and antibacterial resistance [71,72]. Typically, the following bacteria are predominant in bacterial filling of wound surfaces, namely, *Staphylococus aureus*, *Klebsiella terrigena*, *Escherichia coli* and *Pseudomonas aeruginosa* [73]. Since many bacterial strains have become resistant to almost all available antibiotics, the development of novel antimicrobial drugs is of the utmost importance. For example, *R. terrigena* has an antibiotic susceptibility profile with multidrug resistance and a high mortality rate. It can affect various tissues and organs, as well as provoke a general septic infection. *E. coli*, in contrast, is very sensitive to aminoglycoside antibiotics. However, all new virulent strains of *E. coli* that have appeared as a result of mutations, such as the O157:H7 strain, are highly resistant to antibiotics and could have detrimental consequences for the host organisms. Thus, studies concerning the use of OTOA in patch therapy (wound dressings) is of significant importance, since OTOA has proven to be a powerful antibacterial agent [74,75,76].

**Figure 8:**
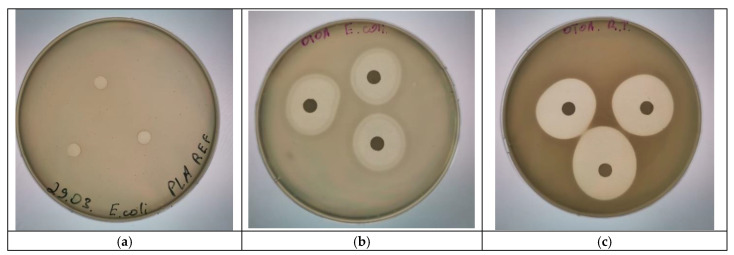
Comparison of antibacterial activity of the reference PLA film against *E. coli* (**a**), the PLA + 50% OTOA film against *E. coli* (**b**) and *R. terrigena* (*Klebsiella terrigena*) (**c**), respectively.

A reference PLA film was used as a negative control and did not show any antibacterial activity against *R. terrigena* and *E. coli* (Figure 8a). Data on the antibacterial activity of pure OTOA were obtained in a previous study [13]. According to the results shown in Figure 8 and Table 5, the PLA+ 50% OTOA circles showed large inhibition zones for both bacterial strains: 29.2 ± 0.4 mm for *R. terrigena* and 27.2 ± 0.2 mm for *E. coli*. The lysis zones remained free of bacterial growth as a result of the antibacterial effect of OTOA, which was maintained over prolonged periods of time (up to several days).

The obtained results confirmed that PLA + 50% OTOA films are promising materials for wound dressings with high antibacterial activity against selected bacterial strains. They support recent reports on the application of various ozonated oils as potent antibacterial agents which could combine wound healing and the ability to overcome drug resistance issues due to the non-specific mechanisms of their antibacterial action [76,77]. One of the proposed mechanisms includes the interaction of ozonides with unsaturated fatty acids in lipid membranes, which leads to an increase in their permeability, membrane disintegration and cell lysis. In addition, ozonated oils could generate reactive oxygen species (ROS) that induce oxidative stress in bacteria. While ozonated oils demonstrate promising antibacterial properties, their efficacy varies based on the type of bacteria, oil source and type of delivery system used [74]. Therefore, further research is needed to optimize their application in terms of increased efficacy, improved physico-chemical properties and safety of products containing ozonated oils.

The controlled release of bioactive compounds and modifiers has emerged as a highly important property considering the implementation of polymeric materials in biomedicine, packaging design, cosmetics and eco-friendly services [78,79,80,81,82,83]. Regarding PLA films and fibers, its moderate crystallinity and sufficient amorphous volume with an appropriate structure could enable efficient drug encapsulation and potential to realize sophisticated kinetic release profiles for innovative pharmaceutical platforms and active barrier materials [84,85]. The results obtained in this study show that PLA + 50% OTOA film could be characterized with sustained OTOA release with a release profile close to zero-order kinetics, which is beneficial when it is necessary to provide a stable drug release pattern. Results on the OTOA release from the PLA matrix were correlated with the changes in the physicochemical properties of PLA + OTOA film as a result of incubation in the saline solution. Thus, the decrease in the OTOA release rate at the prolonged stages of PLA + OTOA film incubation could be correlated with the slow hydrolysis of the PLA polymer matrix as the rate-limiting stage of OTOA release. The developed PLA + 50% OTOA film manifested a strong antibacterial effect against different bacterial strains with multidrug resistance behavior and showed that antibacterial properties of the OTOA-loaded PLA films could be maintained over prolonged periods of time of up to several days. Therefore, such materials could be regarded as promising for various biomedical and packaging applications.

## 4. Conclusions

In this study, the kinetics of oleic acid triglyceride ozonide (OTOA) release from PLA+ OTOA film material during incubation in saline solution was comprehensively investigated. Physicochemical and mechanical properties of the PLA and PLA + OTOA films were studied before and after incubation using DSC, XRD and FTIR spectroscopy. Cumulative OTOA release from the PLA film exceeded 50% after more than 7000 min of incubation in the normal saline. The kinetic profile of OTOA release has two distinct stages, namely, the initial stage (up to 3000 min), characterized by a release process substantially controlled by the relaxation of the PLA matrix, and the second stage above 3000 min with an extremely low rate of OTOA release. The latter appears to be associated with the slow degradation of the PLA polymer and could be approximated by zero-order kinetics. Observed zero-order kinetics provides stable OTOA release with a constant rate from the PLA matrix, which is beneficial for various biomedical applications of OTOA-loaded PLA film materials. The results of DSC and XRD showed that incubation in the saline solution and corresponding OTOA release from the PLA matrix led to the formation of a more disturbed α’ phase of PLA due to the partial hydrolysis of amorphous zones and/or most unstable crystallites in the PLA/OTOA semi-crystalline structure. On the other hand, the degree of crystallinity of PLA + OTOA film was slightly increased at the prolonged stages of incubation, which could be attributed to the enhanced mobility of hydrolyzed PLA chains leading to the formation of additional crystalline structures. According to FTIR spectroscopy, OTOA release from the PLA matrix in saline solution is accompanied by partial destruction of the ozonide structure of OTOA; however, this process does not affect the general kinetics of OTOA release. PLA + 50% OTOA film retained its strength properties after 6100 min of incubation in normal saline, while relative elongation at break was drastically decreased after incubation. This was attributed to the loss of plasticizing agent (OTOA) from the PLA matrix due to OTOA release and partial OTOA hydrolysis. The developed PLA + 50% OTOA film showed pronounced antimicrobial activity against *Raoultella terrigena* (*Klebsiella terrigena*) and *Escherichia coli* bacterial strains. Large inhibition zones both for *R. terrigena* and *E. coli* were maintained over prolonged periods of time, indicating a stable antibacterial effect. The PLA + 50% OTOA film material developed in this study is promising for various applications, including wound dressings, with the sustained release of an antibacterial agent, as well as for antibacterial food packaging.

## Figures and Tables

**Table 1:** Changes in opacity and thickness of PLA films depending on the incubation time in normal saline.

Sample	Opacity (mm^−1^)	Film Thickness (µm)
PLA	0.25 ± 0.03	126.0 ± 3.5
PLA 4100 min	0.83 ± 0.05	123.5 ± 3.5
PLA 6100 min	1.23 ± 0.06	125.0 ± 3.7
PLA + 50% OTOA	0.98 ± 0.04	139.5 ± 2.2
PLA + OTOA 50% 4100 min	1.68 ± 0.05	143.0 ± 2.8
PLA + OTOA 50% 6100 min	2.5 ± 0.07	146.0 ± 3.0

**Table 2:** Characteristic bands in the FTIR spectra for pristine PLA and PLA + OTOA films.

PLA Characteristic Bands, cm^−1^	PLA + 50% OTOA Characteristic Bands, cm^−1^	Characteristic Band Assignment
3100–3620 (observed after hydr.)	3100–3620 (after hydr.)	-OH
2996	2995	-CH_3_ (asim)
2945	-	-CH (sim)
-	2927	-CH_2_ (asim)
2880	-	- CH_3_ stretching
2857 (after hydr.)	2856 (OTOA)	-CH_2_ (sim)
1753	1753	-C=O stretching
-	1540 (after hydr.)	-COO
-	1576 (after hydr.)	-COO
1455	1455	–CH_3_ bending

**Table 3:** Degree of crystallinity (χ) for studied reference PLA and PLA + 50% OTOA films obtained from XRD data.

Sample	χ (XRD), (%)
PLA	35.5
PLA 6100 min NaCl	31.3
PLA + 50% OTOA	24.3
PLA + 50% OTOA 4100 min NaCl	32.3
PLA + 50% OTOA 6100 min NaCl	32.6

**Table 4:** Thermodynamic characteristics of PLA and PLA + 50% OTOA films and the degree of crystallinity (*χ*) of films obtained using DSC.

Sample	T_m_ (°C)	∆H_m_ (J/g)	*β*	*χ* (DSC) (%)
PLA	169.2	39.6	0	42.2
PLA 6100 min	169.6	35.8	0	38.3
PLA + 50% OTOA	159.1 *	11.8 *	0.5	25.2
PLA + OTOA 50% 6100 min	158.3 *	23.9 *	0.25 **	34.0

* Obtained as a result of deconvolution of the overlapping calorimetric peaks. ** Estimated taking into account the 50% release of OTOA from the film during incubation.

**Table 5:** Antibacterial activity of reference PLA film and PLA + 50% OTOA film against *R. terrigena* (*Klebsiella terrigena*) and *E. coli*.

Sample	Bacterial Strain	
	*R. terrigena* (*Klebsiella terrigena*)	*E. coli*
	Size of Clear Zone (mm)	
Reference PLA	0.0 ± 0.0	0.0 ± 0.0
PLA + 50% OTOA	29.2 ± 0.4	27.2 ± 0.2
OTOA *	31.2 ± 0.1	29.0 ± 0.1

* Data obtained from ref. [13].

## Data Availability

The original contributions presented in this study are included in the article. Further inquiries can be directed to the corresponding authors.

## References

[1] Shaili V., Khan S. (2021). A Critical Analysis of the Rising Global Demand of Plastics and its Adverse Impact on Environmental Sustainability. Environ. Pollut. Manag..

[2] Andler R., Tiso T., Blank L., Andreeßen C., Zampolli J., D’Afonseca V., Guajardo C., Díaz-Barrera A. (2022). Current progress on the biodegradation of synthetic plastics: From fundamentals to biotechnological applications. Rev. Environ. Sci. Bio/Technol..

[3] Tasseron P.F., van Emmerik T.H.M., Vriend P., Hauk R., Alberti F., Mellink Y., van der Ploeg M. (2024). Defining plastic pollution hotspots. Sci. Total Environ..

[4] Roy S., Siracusa V. (2023). Multifunctional Application of Biopolymers and Biomaterials. Int. J. Mol. Sci..

[5] Echeverría C., Muñoz-Bonilla A., Cuervo-Rodríguez R., López D., Fernández-García M. (2019). Antibacterial PLA Fibers Containing Thiazolium Groups as Wound Dressing Materials. ACS Appl. Bio Mater..

[6] Alexeeva O., Olkhov A., Konstantinova M., Podmasterev V., Tretyakov I., Petrova T., Koryagina O., Lomakin S., Siracusa V., Iordanskii A.L. (2022). Improvement of the Structure and Physicochemical Properties of Polylactic Acid Films by Addition of Glycero-(9,10-trioxolane)-Trialeate. Polymers.

[7] Ferreira E.F., Mouro C., Silva L., Gouveia I.C. (2022). Sustainable Packaging Material Based on PCL Nanofibers and Lavandula luisieri Essential Oil, to Preserve Museological Textiles. Polymers.

[8] Shao L., Xi Y., Weng Y. (2022). Recent Advances in PLA-Based Antibacterial Food Packaging and Its Applications. Molecules.

[9] Motelica L., Ficai D., Ficai A., Oprea O.C., Kaya D.A., Andronescu E. (2020). Biodegradable Antimicrobial Food Packaging: Trends and Perspectives. Foods.

[10] Siracusa V., Rocculi P., Romani S., Dalla Rosa M. (2008). Biodegradable Polymers for food packaging: A review. Trends Food Sci. Technol..

[11] Olkhov A., Alexeeva O., Konstantinova M., Podmasterev V., Tyubaeva P., Borunova A., Siracusa V., Iordanskii A.L. (2021). Effect of Glycero-(9,10-trioxolane)-trialeate on the Physicochemical Properties of Non-Woven Polylactic Acid Fiber Materials. Polymers.

[12] Rojas A., Velásquez E., Patiño Vidal C., Guarda A., Galotto M.J., López de Dicastillo C. (2021). Active PLA Packaging Films: Effect of Processing and the Addition of Natural Antimicrobials and Antioxidants on Physical Properties, Release Kinetics, and Compostability. Antioxidants.

[13] Alexeeva O.V., Olkhov A.A., Konstantinova M.L., Podmasterev V.V., Petrova T.V., Martirosyan L.Y., Karyagina O.K., Kozlov S.S., Lomakin S.M., Tretyakov I.V. (2024). A Novel Approach for Glycero-(9,10-trioxolane)-Trialeate Incorporation into Poly(lactic acid)/Poly(ε-caprolactone) Blends for Biomedicine and Packaging. Polymers.

[14] Castañeda-Rodríguez S., González-Torres M., Ribas-Aparicio R.M., Del Prado-Audelo M.L., Leyva-Gómez G., Gürer E.S., Sharifi-Rad J. (2023). Recent advances in modified poly (lactic acid) as tissue engineering materials. J. Biol. Eng..

[15] Acik G. (2020). Preparation of antimicrobial and biodegradable hybrid soybean oil and poly (ʟ-lactide) based polymer with quaternized ammonium salt. Polym. Degrad. Stab..

[16] Qin Y., Liu D., Wu Y., Yuan M., Li L., Yang J. (2015). Effect of PLA/PCL/cinnamaldehyde antimicrobial packaging on physicochemical and microbial quality of button mushroom (*Agaricus bisporus*). Postharvest Biol. Technol..

[17] Gutiérrez L., Batlle R., Andújar S., Sánchez C., Nerín C. (2011). Evaluation of antimicrobial active packaging to increase shelf life of gluten-free sliced bread. Packag. Technol. Sci..

[18] Erdohan Z.Ö., Çam B., Turhan K.N. (2013). Characterization of antimicrobial polylactic acid based films. J. Food Eng..

[19] Tokiwa Y., Calabia B.P. (2006). Biodegradability and biodegradation of poly(lactide). Appl. Microbiol. Biotechnol..

[20] Siracusa V. (2019). Microbial Degradation of Synthetic Biopolymers Waste. Polymers.

[21] Omer A.M., Tamer T.M., Khalifa R.E., Eltaweil A.S., Agwa M.M., Sabra S., Abd-Elmonem M.S., Mohy-Eldin M.S., Ziora Z.M. (2021). Formulation and Antibacterial Activity Evaluation of Quaternized Aminochitosan Membrane for Wound Dressing Applications. Polymers.

[22] Singh V., Marimuthu T., Makatini M.M., Choonara Y.E. (2022). Biopolymer-Based Wound Dressings with Biochemical Cues for Cell-Instructive Wound Repair. Polymers.

[23] Mani M.P., Faudzi A.A.M., Ramakrishna S., Ismail A.F., Jaganathan S.K., Tucker N., Rathanasamy R. (2023). Sustainable electrospun materials with enhanced blood compatibility for wound healing applications—A mini review: *Curr*. Opin. Biomed. Eng..

[24] Unnithan A.R., Barakat N.A.M., Tirupathi Pichiah P.B., Gnanasekaran G., Nirmala R., Cha Y.-S., Jung C.-H., El-Newehy M., Kim H.Y. (2012). Wound-dressing materials with antibacterial activity from electrospun polyurethane–dextran nanofiber mats containing ciprofloxacin HCl. Carbohydr. Polym..

[25] Negut I., Grumezescu V., Grumezescu A.M. (2018). Treatment Strategies for Infected Wounds. Molecules.

[26] Subbuvel M., Kavan P. (2021). Development and investigation of antibacterial and antioxidant characteristics of poly lactic acid films blended with neem oil and curcumin. J. Appl. Polym. Sci..

[27] Singh A.A., Sharma S., Srivastava M., Majumdar A. (2020). Modulating the properties of polylactic acid for packaging applications using biobased plasticizers and naturally obtained fillers. Int. J. Biol. Macromol..

[28] Ardjoum N., Chibani N., Shankar S., Fadhel Y.B., Djidjelli H., Lacroix M. (2021). Development of antimicrobial films based on poly(lactic acid) incorporated with Thymus vulgaris essential oil and ethanolic extract of Mediterranean propolis. Int. J. Biol. Macromol..

[29] Altun E., Yuca E., Ekren N., Kalaskar D.M., Ficai D., Dolete G., Ficai A., Gunduz O. (2021). Kinetic Release Studies of Antibiotic Patches for Local Transdermal Delivery. Pharmaceutics.

[30] Smith R., Russo J., Fiegel J., Brogden N. (2020). Antibiotic Delivery Strategies to Treat Skin Infections When Innate Antimicrobial Defense Fails. Antibiotics.

[31] Joy N., Samavedi S. (2020). Identifying Specific Combinations of Matrix Properties that Promote Controlled and Sustained Release of a Hydrophobic Drug from Electrospun Meshes. ACS Omega.

[32] Ramos M., Fortunati E., Beltran A., Peltzer M., Cristofaro F., Visai L., Valente A.J.M., Jimenez A., Kenny J.M., Garrigos M.C. (2020). Controlled Release, Disintegration, Antioxidant, and Antimicrobial Properties of Poly (Lactic Acid)/Thymol/Nanoclay Composites. Polymers.

[33] Iordanskii A.L., Zaikov G.E., Berlin A.A. (2015). Diffusion kinetics of hydrolysis of biodegradable polymers. Weight loss and control of the release of low molecular weight substances. Polym. Sci. Ser. D.

[34] Soares J.S., Zunino P. (2010). A mixture model for water uptake, degradation, erosion and drug release from polydisperse polymeric networks. Biomaterials.

[35] Peppas N.A. (1983). A model of dissolution-controlled solute release from porous drug delivery polymeric systems. J. Biomed. Mater. Res..

[36] Sevim K., Pan J. (2018). A model for hydrolytic degradation and erosion of biodegradable polymers. Acta Biomater..

[37] Feldstein M.M., Raigorodskii I.M., Iordanskii A.L., Hadgraft J. (1998). Modeling of percutaneous drug transport in vitro using skin-imitating Carbosil membrane. J. Control. Release.

[38] Iordanskii A., Karpova S., Olkhov A., Borovikov P., Kildeeva N., Liu Y. (2019). Structure-morphology impact upon segmental dynamics and diffusion in the biodegradable ultrafine fibers of polyhydroxybutyrate-polylactide blends. Eur. Polym. J..

[39] Lomakin S., Mikheev Y., Usachev S., Rogovina S., Zhorina L., Perepelitsina E., Levina I., Kuznetsova O., Shilkina N., Iordanskii A. (2024). Evaluation and Modeling of Polylactide Photodegradation under Ultraviolet Irradiation: Bio-Based Polyester Photolysis Mechanism. Polymers.

[40] Crank J. (1975). The Mathematics of Diffusion.

[41] Siepmann J., Peppas N.A. (2011). Higuchi equation: Derivation, applications, use and misuse. Int. J. Pharm..

[42] Korsmeyer R.W., Gurny R., Doelker E., Buri P., Peppas N.A. (1983). Mechanisms of solute release from porous hydrophilic polymers. Int. J. Pharm..

[43] Zhu W., Long J., Shi M. (2023). Release Kinetics Model Fitting of Drugs with Different Structures from Viscose Fabric. Materials.

[44] Han J.H., Floros J.D. (1997). Casting Antimicrobial Packaging Films and Measuring Their Physical Properties and Antimicrobial Activity. J. Plast. Film Sheeting.

[45] Li S., McCarthy S. (1999). Further investigations on the hydrolytic degradation of poly (DL-lactide). Biomaterials.

[46] Balouiri M., Sadiki M., Ibnsouda S.K. (2016). Methods for in vitro evaluating antimicrobial activity: A review. J. Pharm. Anal..

[47] Murray P.R., Jo E., Turnidge B. (2007). Manual of Clinical Microbiology.

[48] Clinical and Laboratory Standards Institute (CLSI) (2012). Performance Standards for Antimicrobial Disk Susceptibility Tests; Approved Standard.

[49] Clinical and Laboratory Standards Institute (CLSI) (2009). Method for Antifungal Disk Diffusion Susceptibility Testing of Yeasts; Approved Guideline.

[50] Rothstein S.N., Federspiel W.J., Little S.R. (2009). A unified mathematical model for the prediction of controlled release from surface and bulk eroding polymer matrices. Biomaterials.

[51] Masters D.B., Berde C.B., Dutta S., Turek T., Langer R. (1993). Sustained local anesthetic release from bioerodible polymer matrices: A potential method for prolonged regional anesthesia. Pharm. Res..

[52] Laracuente M.-L., Yu M.H., McHugh K.J. (2020). Zero-order drug delivery: State of the art and future prospects. J. Control. Release.

[53] Song Z., Li X., Wu K., Cai S. (2023). Nonequilibrium thermodynamic modeling of case II diffusion in glassy polymers. J. Mech. Phys. Solids.

[54] Park K., Skidmore S., Hadar J., Garner J., Park H., Otte A., Soh B.K., Yoon G., Yu D., Yun Y. (2019). Injectable, long-acting PLGA formulations: Analyzing PLGA and understanding microparticle formation. J. Control. Release.

[55] Dekyndt B., Verin J., Neut C., Siepmann F., Siepmann J. (2015). How to easily provide zero order release of freely soluble drugs from coated pellets. Int. J. Pharm..

[56] Li S., Garreau H., Vert M. (1990). Structure-property relationships in the case of the degradation of massive poly(α-hydroxy acids) in aqueous media. J. Mater. Sci. Mater. Med..

[57] Mengjiao Z., Sitong Y., Qingxiu J. (2019). Degradation Performance of Electrospun Polylactic Acid/Cellulose Nanocrystalline Composite Fiber Membrane. Journal of Physics: Conference Series.

[58] Qin L., Qiu J., Liu M., Ding S., Shao L., Lü S., Zhang G., Zhao Y., Fu X. (2011). Mechanical and thermal properties of poly(lactic acid) composites with rice straw fibermodified by poly(butyl acrylate), *Chem*. Eng. J..

[59] Chen C., Chueh Y., Tseng H., Huang M., Lee Y. (2003). Preparation and characterization of biodegradable PLA polymeric blends. Biomaterials.

[60] Qu P., Gao Y., Wu G., Zhang L. (2010). Nanocomposite of poly(lactid acid) reinforced with cellulose nanofibrils. BioResources.

[61] Chieng B.W., Ibrahim N.A., Then Y.Y., Loo Y.Y. (2014). Epoxidized Vegetable Oils Plasticized Poly(lactic acid) Biocomposites: Mechanical, Thermal and Morphology Properties. Molecules.

[62] Moliner C., Finocchio E., Arato E., Ramis G., Lagazzo A. (2020). Influence of the Degradation Medium on Water Uptake, Morphology, and Chemical Structure of Poly(Lactic Acid)-Sisal Bio-Composites. Materials.

[63] Farid T., Herrera V.N., Kristiina O. (2018). Investigation of crystalline structure of plasticized poly (lactic acid)/Banana nanofibers composites. IOP Conf. Ser. Mater. Sci. Eng..

[64] Song Y., Tashiro K., Xu D., Liu J., Bin Y. Crystallization behavior of poly(lactic acid)/microfibrillated cellulose composite, Polymer 2013, 54, 3417–3425.

[65] Pan P., Zhu B., Kai W., Dong T., Inoue Y. (2008). Polymorphic Transition in Disordered Poly(l-lactide) Crystals Induced by Annealing at Elevated Temperatures. Macromolecules.

[66] Luo Y., Lin Z., Guo G. (2019). Biodegradation Assessment of Poly (Lactic Acid) Filled with Functionalized Titania Nanoparticles (PLA/TiO_2_) under Compost Conditions. Nanoscale Res. Lett..

[67] Giuliana G., Roberto P. (2013). Effect of PLA grades and morphologies on hydrolytic degradation at composting temperature: Assessment of structural modification and kinetic parameters. Polym. Degrad. Stab..

[68] Kolstad J. (1996). Crystallization kinetics of poly(L-lactideco-meso-lactide). J. Appl. Polym. Sci..

[69] Ljungberg N., Wesslén B. (2002). The effects of plasticizers on the dynamic mechanical and thermal properties of poly(lactic acid). J. Appl. Polym. Sci..

[70] Inácio E.M., Lima M.C.P., Souza D.H.S., Sirelli L., Dias M.L. (2018). Crystallization, thermal and mechanical behavior of oligosebacate plasticized poly(lactic acid) films. Polymer.

[71] Lekhniuk N., Fesenko U., Pidhirnyi Y., Sekowska A., Korniychuk O., Konechnyi Y. (2021). Raoultella terrigena: Current state of knowledge, after two recently identified clinical cases in Eastern Europe. Clin. Case Rep..

[72] Poirel L., Madec J.Y., Lupo A., Schink A.K., Kieffer N., Nordmann P., Schwarz S. (2018). Antimicrobial Resistance in *Escherichia coli*. Microbiol. Spectr..

[73] Bao L., Peng R., Ren X., Ma R., Li J., Wang Y. (2013). Analysis of some common pathogens and their drug resistance to antibiotics. Pak. J. Med. Sci..

[74] Ugazio E., Tullio V., Binello A., Tagliapietra S., Dosio F. (2020). Ozonated Oils as Antimicrobial Systems in Topical Applications. Their Characterization, Current Applications, and Advances in Improved Delivery Techniques. Molecules.

[75] Moureu S., Violleau F., Haimoud-Lekhal D.A., Calmon A. (2015). Ozonation of sunflower oils: Impact of experimental conditions on the composition and the antibacterial activity of ozonized oils. Chem. Phys. Lipids.

[76] de Almeida Kogawa R.N., de Arruda E.J., Micheletti A.C., Cepa Matos M.d.F., Silva de Oliveira L.C., de Lima D.P., Pereira Carvalho N.C., de Oliveira P.D., de Castro Cunha M., Ojeda M. (2015). Synthesis, characterization, thermal behavior and biological activity of ozonides from vegetable oils. RSC Adv..

[77] Puxeddu S., Scano A., Scorciapino M.A., Delogu I., Vascellari S., Ennas G., Manzin A., Angius F. (2024). Physico-Chemical Investigation and Antimicrobial Efficacy of Ozonated Oils: The Case Study of Commercial Ozonated Olive and Sunflower Seed Refined Oils. Molecules.

[78] Kalın G., Alp E., Chouaikhi A., Roger C. (2023). Antimicrobial Multidrug Resistance: Clinical Implications for Infection Management in Critically Ill Patients. Microorganisms.

[79] Han S., Fisher J.P., Mikos A.G., Hogan K.J. (2024). Polymeric nanomaterials in 3D bioprinting for tissue engineering and drug delivery applications. Bioprinting.

[80] Puri S., Mazza M., Roy G., England R.M., Zhou L., Nourian S., Subramony J.A. (2023). Evolution of nanomedicine formulations for targeted delivery and controlled release. Adv. Drug Deliv. Rev..

[81] Dong Y., Jiang T., Wu T., Wang W., Xie Z., Yu X., Peng Y., Wang L., Xiao Y., Zhong T. (2024). Enzyme-responsive controlled-release materials for food preservation and crop protection—A review. Int. J. Biol. Macromol..

[82] Ćorović M., Milivojević A., Simović M., Banjanac K., Pjanović R., Bezbradica D. (2020). Enzymatically derived oil-based L-ascorbyl esters: Synthesis, antioxidant properties and controlled release from cosmetic formulations. Sustain. Chem. Pharm..

[83] Zhao S., Li L., Wang Y., Liu Z., Yang S., Gao X., Zhang C., Yu A. (2024). Remediation of petroleum-contaminated site soil by bioaugmentation with immobilized bacterial pellets stimulated by a controlled-release oxygen composite. Environ. Pollut..

[84] Zare I., Chevrier D.M., Cifuentes-Rius A. (2023). Protein-protected metal nanoclusters as diagnostic and therapeutic platforms for biomedical applications. Mater. Today.

[85] Raghuvanshi S., Khan H., Saroha V., Sharma H., Gupta H.S., Kadam A., Dutt D. (2023). Recent advances in biomacromolecule-based nanocomposite films for intelligent food packaging—A review. Int. J. Biol. Macromol..

